# Colorimetric Sensors for Chemical and Biological Sensing Applications

**DOI:** 10.3390/s23052749

**Published:** 2023-03-02

**Authors:** Yu Wu, Jing Feng, Guang Hu, En Zhang, Huan-Huan Yu

**Affiliations:** 1 School of Pharmacy and Bioengineering, Chongqing University of Technology, Chongqing 400054, China; 2 Chongqing Institute for Food and Drug Control, Chongqing 401121, China; 3 College of Pharmaceutical Sciences, Southwest University, Chongqing 400715, China

**Keywords:** colorimetric, nanomaterials, LSPR, nanozyme, chemical sensor, biosensor

## Abstract

Colorimetric sensors have been widely used to detect numerous analytes due to their cost-effectiveness, high sensitivity and specificity, and clear visibility, even with the naked eye. In recent years, the emergence of advanced nanomaterials has greatly improved the development of colorimetric sensors. This review focuses on the recent (from the years 2015 to 2022) advances in the design, fabrication, and applications of colorimetric sensors. First, the classification and sensing mechanisms of colorimetric sensors are briefly described, and the design of colorimetric sensors based on several typical nanomaterials, including graphene and its derivatives, metal and metal oxide nanoparticles, DNA nanomaterials, quantum dots, and some other materials are discussed. Then the applications, especially for the detection of metallic and non-metallic ions, proteins, small molecules, gas, virus and bacteria, and DNA/RNA are summarized. Finally, the remaining challenges and future trends in the development of colorimetric sensors are also discussed.

## 1. Introduction

Sensing techniques have been wildly applied in a variety of areas, including medicine and health, environmental science, and the food industry. Traditional sensing techniques (such as traditional electronic, electrochemical, fluorescent, and optical sensors) are composed of complex systems. Even though they can satisfy the detection of various analytes, the obvious drawbacks may hinder their practical applications. For example, traditional sensing techniques always need costly instruments, skilled manpower, and proper sampling and storage. In addition, traditional sensing techniques also require complex pretreatment processes, and the results can’t be observed immediately, which is not convenient for the clinical diagnosis of diseases. Furthermore, for the detection of pollutants and biomarkers, traditional sensing techniques have their limitations when the concentration to be measured is at the ppm level. Traditional sensing techniques, based on organic dyes are not as sensitive as colorimetric sensors, and they can’t determine individual components of a mixture without employing chemometric methods. On the contrary, colorimetric sensors are extremely favored because of their cost-effectiveness and high sensitivity and specificity. In particular, the results obtained with colorimetric sensors can be easily judged through naked-eye observation without sophisticated instruments [1]. In the past decade, colorimetric sensors have been used for the detection of chemical compounds, organic pollutants, heavy metal ions, and biomolecules [2,3,4].

With the development of nanotechnology and nanomaterials, researchers have paid considerable attention to the design and fabrication of various functional materials for sensing, such as graphene and its derivatives [5,6], metal nanoparticles [7,8], metal oxide nanoparticles [9,10], quantum dots [11,12], DNA nanomaterials [13,14], and other materials. Additionally, colorimetric sensors based on reactions between organic dyes and target analytes have been proposed for detection. However, the application of this colorimetric method is quite limited because of the low extinction coefficients of organic dyes, which made them unable to perform at a higher sensitivity [15]. To overcome this deficiency, label-free nanozymes, metal nanoparticles, and other types of colorimetric sensors have been designed and applied to detect target analytes. Rostami et al. [16] demonstrated the intrinsic peroxidase-like activity of graphene nanoribbons (GNR) which were employed as a label-free nanozyme for the rapid colorimetric detection of dopamine (DA). Qiu et al. [17] developed gold nanoparticles (Au NPs) based colorimetric sensor which was used to detect metal ions. Yin et al. [18] reported a bimetallic nanoparticles-based colorimetric sensor for the detection of disease biomarkers in clinical samples.

Since the development of colorimetric sensors is fast, especially in recent years, it is necessary to summarize the updated information systematically. In this review, we focused on the recent (2015–2022) advances in colorimetric sensors and describe the classifications, sensing mechanisms, and fabrication of high-performance colorimetric sensors including graphene and its derivatives, metal and metal oxide nanoparticles, quantum dots, DNA, and other materials. Furthermore, the applications of the colorimetric sensors on the detection of drugs, chemical compounds, biomolecules, pollutants, and others are shown in typical cases. Finally, we discuss the problems in the current stage of application and provide some perspectives on the future trends of colorimetric sensors.

## 2. Discussion

### 2.1. Classifications of Colorimetric Sensors

Colorimetric sensing is highly related to the materials which are used to fabricate sensors. Colorimetric sensors enable detection by using nanozyme or nanozyme-like materials to catalyze the chemical reactions that accompany color changes. Moreover, colorimetric recognition of chemical or biological analytes can also be realized by the unique localized surface plasmon resonance (LSPR) properties of metal nanoparticles. Due to the excellent properties of graphene and its derivatives, metal and metal oxide nanoparticles, DNA nanomaterials, and other types of carbon-based materials, in this section, we present these materials for fabricating colorimetric sensors.

#### 2.1.1. Graphene and Its Derivatives-Based Colorimetric Sensors

Graphene and its derivatives have shown great potential for sensing due to their extraordinary physicochemical, optical, and electrical properties. Graphene has a flat 2D nanostructure with high a surface area, which can provide an extremely high density of surface-active sites for binding analytes to enhance the sensitivity of colorimetric sensors [19,20]. Nowadays, various methods have been developed for preparation of graphene and its derivatives. Mechanical exfoliation, chemical vapor deposition, epitaxial growth, and other methods are commonly used to prepare graphene, Hummer’s method is the classical method for graphene oxide (GO) preparation, and modified Hummer’s method and microwave-assisted method have also been used for the preparation of GO [19]. Reduced graphene oxide (RGD) is much easier to prepare than graphene and GO, and RGD can be prepared from GO by reductant reagents. Alternatively, the photocatalytic, electrochemical, microwave, and thermal methods have been used to prepare RGO [21]. At present, the synthesis of graphene quantum dots (GQDs) mainly include top-down methods (such as hydrothermal/solvothermal, liquid phase exfoliation, and electrochemical exfoliation of graphene) and down-top methods (such as solution chemical synthesis, microwave synthesis, and cage opening of fullerenes) [22]. Graphene and its derivatives also have broadband light absorption, and strong polarization-dependent effects, thus they have been successfully applied to construct a visual detection platform that can be used for determining various analytes like small molecules, DNA, pollutants, biomolecules, and so on [23].

**Figure 1:**
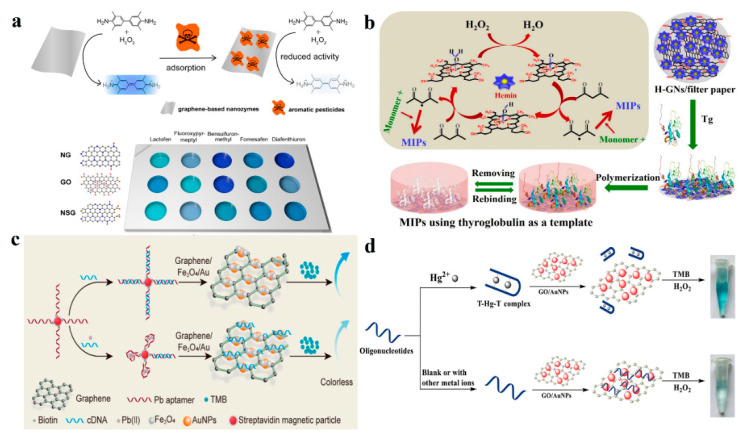
(**a**) Nanozyme sensor arrays based on heteroatom-doped graphene for detecting pesticides. Reprinted with permission from Ref. [28]. Copyright 2020 ACS; (**b**) The fabrication principle of H–GNs paper-based colorimetric sensor. Reprinted with permission from Ref. [32]. Copyright 2019 Elsevier. (**c**) Principle of the detection of Pb^2+^ on graphene/Fe_3_O_4_-Au NPs sensing platform. Reprinted with permission from Ref. [34]. Copyright 2020 MDPI. (**d**) Schematic illustration of oligonucleotide-based colorimetric sensor for Hg^2+^ detection using smart interface of GO/Au NPs. Reprinted with permission from Ref. [35]. Copyright 2021 Elsevier.

Nanozyme sensors have attracted increasing attention due to their low cost and stability over natural enzymes [24]. With the development of nanomaterials, researchers have proved that nanomaterials have catalytic activity when used alone or hybridized with other nanomaterials. For example, graphene and its derivatives with peroxidase-like activity can react with 3,3′,5,5′-tetramethylbenzidine (TMB) in the presence of H_2_O_2_ accompanying the color changes [25,26]. For example, Ali et al. [16] reported graphene nanoribbons (GNR)-based colorimetric sensors for the detection of dopamine. In this article, the authors investigated the catalytic activity of GNR, GO, and multi-walled carbon nanotubes (MWCNT), and the results demonstrated an enhanced intrinsic peroxidase-like activity of GNR over GO and MWCNT. This may be due to the smaller size and higher rate of electron transfer of nanoribbons than graphene sheets, therefore, accelerating the redox reaction between TMB and H_2_O_2_ [27]. Wei’s group developed [28] heteroatom-doped graphene-based nanozyme sensor arrays for aromatic pesticides detection (Figure 1a). In the presence of pesticides, the peroxidase-mimicking activity of nanozyme could be decreased when the active sites on graphene were masked by adsorption, followed by the reduced blue color. Furthermore, this proposed nanozyme sensor array can also discriminate different pesticides with different concentrations. He and coworkers [29] synthesized L-cysteine functionalized graphene oxide nanosheets (CGO) for simultaneous enrichment and a colorimetric assay of trace mercury ions. It was found that CGO exhibited higher peroxidase-like catalytic activity than the pristine graphene oxide (GO), due to the abundant S and N-containing active sites on CGO, which could improve the catalytic activity. In addition, the S-containing groups on CGO had a strong affinity to Hg^2+^, and the loading of Hg^2+^ could dramatically enhance the peroxidase-like activity of CGO. This was because the sensing principle was based on the competitive adsorption between TMB and Hg^2+^ over CGO. The Hg^2+^ occupied the active sites which hindered the TMB binding on CGO, resulting in an increase of free TMB in the solution which could be oxidized by ·OH to produce colored oxidation products. GQDs with a side less than 100 nm in lateral dimension, have not only been employed as probes due to their satisfactory optical properties but also served as a nanozyme for their intrinsic peroxidase-like activity [30]. Bi et al. [31] employed terephthalic acid-modified graphene quantum dots (TPA@GQDs) as a catalyst, and different metal ions (Fe^2+^, Cu^2+^, and Zn^2+^) were integrated with TPA@GQDs to regulate the catalytic activity due to the combination between metal ions and hydroxyl groups on the surface of TPA@GQDs. The catalytical activities of this sensing platform were determined by the affinity between metal ions and analytes. As we know, metal ions preferentially combine with mercaptan over hydroxyl groups, therefore, this sensing platform can show diverse absorbance changes by the unique colorimetric response to corresponding thiols. Moreover, this sensor array has been used for diagnosing cancer due to the various levels of GSH in different types of cells.

Some researchers found that hemin and metal or metal oxide nanoparticles modified graphene and its derivatives exhibited intrinsic peroxidase-like catalytic activity, and served as artificial enzymes for detecting metal ions, small molecules, and biological molecules. For example, Jing and coworkers [32] prepared molecular imprinting polymers (MIP) on the surface of hemin–graphene nanosheets (H–GNs) for the fabrication of the colorimetric paper-based sensor for high molecular weight protein detection (Figure 1b). The roles of H–GNs were the supporters to enrich high molecular weight protein, the initiators for the preparation of MIP film, and the catalytic enzymes for the oxidation of TMB. The superior adsorption capacity and imprinting factors made the functional paper-based colorimetric sensor exhibit a highly selective recognition ability for thyroglobulin. Lu et al. [33] synthesized histidine-capped gold nanoclusters (His@AuNCs) with intrinsic oxidase-like activity, which could directly oxidize TMB to produce blue-colored ox-TMB in the absence of H_2_O_2_. His@AuNCs were further combined with RGO, and the catalytic activity of His@AuNCs/RGO nanocomposites increased dramatically. Furthermore, the oxidase-like activity of the as-prepared His@AuNCs/RGO was evaluated with nitrite and TMB as substrates. The results demonstrated that nitrite could inhibit the catalytic activity of His@AuNCs/RGO in the oxidation of TMB because TMB and nitrite may share the same catalytic active sites. According to this finding, His@AuNCs/RGO was used as an oxidase mimic for the detection of nitrite in a spectrophotometric sensor.

Later on, to endow graphene-based nanozyme sensors with remarkable selectivity, the coupling of the graphene nanocomplexes with aptamers has been proposed to satisfy the increasing demands. Wang et al. [34] fabricated a colorimetric aptamer sensor based on graphene/Fe_3_O_4_-Au NPs to enhance the peroxidase activity for Pb^2+^ detection. The principle of this sensing platform is shown in Figure 1c. In the absence of Pb^2+^, magnetic beads with aptamer combined with cDNA, and graphene/Fe_3_O_4_-Au NPs would exert high activity for catalyzing TMB to a blue color. In the presence of Pb^2+^, the aptamer of Pb^2+^ preferred to bind Pb^2+^ and inhibited the catalytic activity of the sensing platform by leaving a large number of cDNA in a free state which would then adsorb onto the surface of graphene/Fe_3_O_4_-Au NPs to occupy the active catalytic sites. Chen and colleagues [35] developed a colorimetric sensor based on graphene oxide (GO)/Au NPs nanocomposites combined with the high specificity of oligonucleotide aptamers that can recognize ultra-low mercury ions via thymine–Hg^2+^–thymine interaction (Figure 1d). The specific T–Hg^2+^–T binding made the Hg^2+^ easy to be selectively detected by the proposed colorimetric sensor.

In this section, we summarize the studies of colorimetric sensors based on graphene and its derivatives. Although these colorimetric sensors worked on different principles, they were all able to accurately identify biomolecules, pesticides, metal ions, small molecules, with wide detection range and low detection limits as presented in Table 1.

#### 2.1.2. Metal and Metal Oxide Nanoparticles Colorimetric Sensors

Metal and metal oxide nanoparticles, especially gold- and silver-nanoparticles and ferroferric oxide/sulfide nanoparticles, have been wildly used as the ideal candidates for developing highly sensitive sensors due to their unique optical, chemical, and magnetic properties; excellent catalytic activity; controlling size; and easy modification [3]. The synthesis of metal and metal oxide nanoparticles are flexible. Similar to the synthesis of GQDs, gold- and silver-nanoparticles also can be synthesized by top-down and down-top methods. In top-down methods, the destructive chemical–physical methods (such as grinding, milling, or physical vapor deposition) have been used to reduce bulk materials to smaller dimensions. In down-top methods, a soluble salt is dissolved in solution, and then the metal center must be reduced to valence 0 by various reductant reagents [36]. Metal oxides nanoparticles can be prepared by several routes, such as hydrothermal, solvothermal, co-precipitation, microemulsion synthesis, sol-gel methods, etc. [37,38].

After the pioneering report on the peroxidase-mimic activity of ferromagnetic nanoparticles [39], the nanozyme-like property of Au NPs [17], silver nanoparticles (Ag NPs) [40], and other magnetic metal oxide or sulfide nanoparticles have been developed [41,42]. Compared with natural enzymes, these nanozymes had several advantages, including excellent catalytic activity, long-term stability, facial synthesis, and surface functionalization. In addition, the catalytic activity of the as-prepared nanomaterials could be adjusted by controlling their size, constructing hybrids, coating the surface with active additions, and selecting compositions [18,43,44,45,46]. Furthermore, the unique localized surface plasmon resonance (LSPR) properties of metal nanoparticles, related to their characteristic colors, dispersion, and aggregation status, shape, and size, made them promising for colorimetric recognition of chemical or biological analytes [47,48].

##### Metal and Metal Oxide Nanoparticles Based on Nanozymes-like Characteristics

**Figure 2:**
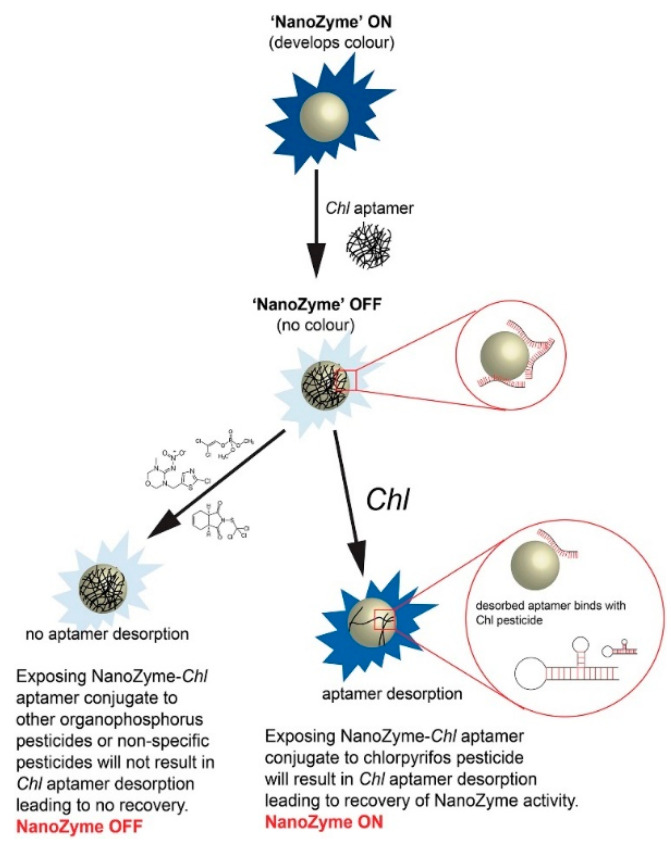
The working principle for chlorpyrifos aptasensor. Reprinted with permission from Ref. [51]. Copyright 2019 Elsevier.

Among the various types of metal and metal oxide nanoparticles, Au NPs have attracted various attention due to their excellent optical properties, and have been extensively employed as colorimetric probes for various analytes detection [47,48]. The colorimetric sensing theory of metal and metal oxide-based nanozyme was similar to graphene-nanozymes, which indicated that these nanozymes can catalyze the oxidation of substrate TMB or 2,2-azino-bis(3-ethylbenzothiazoline-6-sulfonic acid) (ABTS) in the presence of H_2_O_2_ to generate blue color in aqueous solutions. For example, Sun et al. [49] fabricated histidine-protected gold nanoclusters (His-AuNCs)-based colorimetric on–off–on switch for sensing phosphate-containing metabolites and ALP. TMB, ABTS, and o-phenylenediamine (OPD) were used to investigate the catalytic activity and principle of His-AuNCs. The results showed that phosphate-containing metabolites, including PPi, ATP, and ADP, could inhibit the activity of His-AuNCs by obstructing the superoxide radical (O_2_·^−^) generation and electron transfer processes. When alkaline phosphatase (ALP) was introduced into the sensing system, ALP could hydrolyze PPi, ATP, and ADP to yield Pi and adenosine within 30 min, leading to the retrieval of AuNCs catalytic ability. Wang and coworkers [50] proposed a label-free sensing strategy based on the nanozyme property of glutathione–Ag nanoparticles (GSH–Ag NPs) for colorimetric detection of vitamin B1 (VB1). When H_2_O_2_ was presented, the GSH–Ag NPs-TMB system showed a blue color, indicating TMB was oxidized to ox-TMB. It could also be demonstrated by the change in absorption spectra. The excellent peroxidase-like activity of GSH–Ag NPs was possibly attributed to the specific interaction between the TMB and –SH group on the surface of Ag NPs, which could enhance the affinity of Ag NPs towards TMB. Furthermore, GSH–Ag NPs could catalyze H_2_O_2_ to produce ·OH, leading to the oxidation of TMB to produce the characteristic color. When VB1 was added into the GSH–Ag NPs system, the peroxidase-like activity of GSH–Ag NPs was dramatically decreased. Most likely, the negatively charged GSH–Ag NPs combined with the positively charged VB1 via electrostatic interaction. Electrostatic interaction can neutralize the surface charge and lead to aggregation, decreasing the available active sites of GSH–Ag NPs. In addition, it could also reduce the attraction of GSH–Ag NPs toward TMB. Ramanathan’s group [51] constructed a new colorimetric aptasensor for the detection of organophosphorus pesticides based on tyrosine-capped silver nanoparticles modified with chlorpyrifos-specific aptamer (*Chl*) via non-covalent interaction. To evaluate the peroxidase-like activity of Ag NPs, the catalytic oxidation of TMB, ABTS, and OPD (chromogenic substrates) in the presence of H_2_O_2_ was tested. The results illustrated that the catalytic oxidation capacity of Ag NPs for TMB was higher than the other two peroxidase substrates, due to their strong electrostatic interaction and the ability of Ag NPs to cleave the leaving group in TMB. Then, *Chl* was used as a molecular recognition element, which could bind to Ag NPs through non-covalent interaction for target-specific chlorpyrifos. As shown in Figure 2, Ag NPs with intrinsic peroxidase-like activity could convert colorless TMB substrate to a blue product (“NanoZyme” ON). After the co-incubation of Ag NPs and *Chl*, a *Chl*-Ag NPs sensor probe was formed where *Chl* passivated the surface of Ag NPs through non-covalent interactions resulting in the loss of nanozyme activity (“NanoZyme” OFF). When the sensor probe was exposed to non-specific pesticides or other non-specific organophosphorus pesticides, no color was produced, indicating that the aptamers were still bound to the sensor probe (“NanoZyme” OFF). On the contrary, in the presence of chlorpyrifos, the high affinity of *Chl* to chlorpyrifos led to the dissociation of *Chl* from the sensor probe, resulting in a recovery of nanozyme activity (“NanoZyme” ON). According to the literature, the number of *Chl* desorbed from the sensor probe depended on the concentration of chlorpyrifos in the samples.

**Figure 3:**
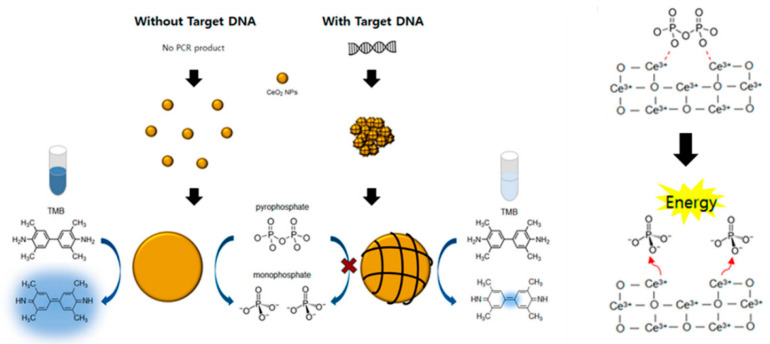
Schematic illustration of CeO_2_ NPs-based colorimetric detection of target DNA using PPi as an enhancer. Reprinted with permission from Ref. [53]. Copyright 2021 MDPI.

Moon and coworkers [52] developed histidine and other amino-acids-coated magnetic nanoparticles (His@MNPs), and the catalytic activity of the as-prepared materials was investigated via the peroxidase-facilitated oxidation of colorimetric reagent TMB as the substrate with H_2_O_2_. The results illustrated that His@MNPs exhibited about a 10-fold higher catalytic activity than pristine MNPs and other amino-acid-capped MNPs. According to the research, His@MNPs modified with choline oxidase and acetylcholine esterase could be used to detect choline and acetylcholine levels in clinical samples. Therefore, His@MNPs could be used as peroxidase-mimicking nanozymes for the detection of some important biological and clinical targets. Even though magnetic nanoparticles exhibited excellent catalytic activity, they still required a relatively long reaction time to produce the colorimetric signal. In addition, magnetic nanoparticles-based colorimetric sensors for nucleic acids detection still had not achieved satisfactory sensitivity and reproductivity. The colorimetric signal generated by H_2_O_2_ may cause some toxicity issues. To overcome these shortages, Ayemeh et al. [53] designed a cerium oxide nanoparticles-based colorimetric sensor for target nucleic acids detection by the oxidase activity of cerium oxide nanoparticles (CeO_2_ NPs) (Figure 3). The results demonstrated that pyrophosphate (PPi) could effectively improve the oxidase activity of CeO_2_ NPs accompanied with colorimetric signals. However, the PPi-enhanced colorimetric signals of CeO_2_ NPs were suppressed by the addition of target nucleic acids. Most probably, the negatively charged target nucleic acids could combine with the positively charged CeO_2_ NPs through electrostatic interaction, reducing the effective surface area for the interaction with PPi and TMB substrate. In addition, some researchers demonstrated that CeO_2_ NPs exhibited phosphatase-like activity which could hydrolyze phosphate ester bonds in PPi to enhance the colorimetric signals. This facile colorimetric sensor with high selectivity and sensitivity could be used for the detection of nucleic acid biomarkers in point-of-care settings.

##### Metal Nanoparticles Based on Localized Surface Plasmon Resonance

**Figure 4:**
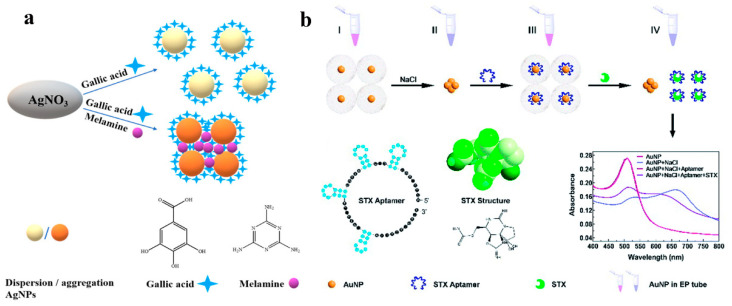
(**a**) Mechanism assay for melamine detection by Ag NPs@galic acid system. Reprinted with permission from Ref. [57]. Copyright 2021 ACS; (**b**) The detection scheme of aptamer-Au NPs for saxitoxin detection. Reprinted with permission from Ref. [60]. Copyright 2020 RSC.

The unique LSPR properties of metal nanoparticles made them ideal for sensitive colorimetric recognition of several analytes [47]. In the colorimetric sensors based on interparticle distance-dependent principles, the aggregation and dispersion of metal nanoparticles induced by analytes could cause an evident color change due to the effect of LSPR [54]. According to the literature, a colloidal solution of 20 nM Au NPs showed a wine-red color, and the LSPR band of this solution occurred at a wavelength of 520 nm. A large red-shift of the LSPR peak was attributed to the aggregation of metal with a characteristic color change from red to blue [55]. Based on the enhanced LSPR optical property, metal nanoparticles attracted considerable attention in the field of colorimetric sensing. For example, Bai and coworkers [56] reported an Au NPs-based colorimetric assay for the detection of SeCys in the presence of Cu^2+^. The proposed SeCys sensing process mainly consisted of two steps: first, the oxidation of Cys by the dissolved oxygen under Cu^2+^ catalysis in the pre-reaction, which can eliminate the interference of Cys; second, the aggregation of Au NPs induced by SeCys and the complex formation of Cu^2+^-SeCys, which led to the color change of Au NPs. This colorimetric sensor had an excellent specificity for SeCys coexisting with cysteine (Cys) and achieved a linear range from 2 μM to 14 μM with a LOD of 0.14 μM. Liu et al. [57] fabricated a simple and specific Ag NPs-based colorimetric sensing assay for the detection of melamine, which depended on the redox reaction of gallic acid with Ag^+^. Due to the formation of hydrogen bonds between gallic acid and melamine, the synthetic monodispersed Ag NPs could aggregate in the presence of melamine, and its characteristic color changed from bright yellow to brown (Figure 4a).

In addition, to improve the sensitivity and selectivity of the colorimetric sensors, Au NPs-based composites could also be promising materials for colorimetric sensing. For example, Lu et al. [58] successfully prepared a porous chitosan/partially reduced graphene oxide/diatomite (CS/prGO/DM), which was combined with Au NPs for a colorimetric assay of pesticides in tea. Firstly, porous CS/prGO/DM was used as the solid phase extraction (SPE) column to concentrate the pesticides in tea. Secondly, Au NPs were employed to assay the concentration of pesticides in the purified tea. Because of the aggregation of Au NPs induced by pesticides, the color of Au NPs changed from red to blue. The G/R values and UV-vis spectra were used for pesticide quantification, and the results showed that the proposed sensing platform could accurately detect phosalone and thiram. As we know, the interferents in complex substrates had a strong influence on the aggregation of Au NPs. CS/prGO/DM could effectively remove different types of tea interferents to address the great challenges of pesticide detection by Au NPs.

To further explore the application of gold nanoparticles (Au NPs), Au NPs were modified with specific molecules, which could specifically react with target molecules, and then became dissociated from Au NPs, leading to the aggregation of Au NPs to induce a color change. For instance, Zhao et al. [59] fabricated a simple highly sensitive and specific aptasensor for the determination of microcystin-LR (MC-LR). The aptamer with a certain base sequence, which could bind MC-LR with high affinity, was introduced into the Au NPs system, and it could be absorbed on the surface of Au NPs via the coordination interaction between the N atom of bases and Au NPs. After modification with aptamer, owing to the more negative charges, Au NPs would be more stable and dispersed against salt-induced aggregation. When MC-LR was added into the aptamer–Au NPs solution, aptamer would specifically react with MC-LR, resulting in the change of color from wine to violet-blue induced by the aggregation of Au NPs. Furthermore, this aptasensor could not be disturbed by the coexisting substances, and Au NPs could still maintain the dispersive state even in a concentrated salt solution. Han and coworkers [60] developed an aptamer-functionalized Au NPs-based colorimetric biosensor for saxitoxin detection (Figure 4b). This aptamer could selectively react with saxitoxin, resulting in the aggregation of Au NPs, and the change of color from red to blue-purple as the concentration of saxitoxin increased. This aptamer–Au NPs-based biosensor had a linear relationship with the concentration of saxitoxin in the range of 10 fM–0.1 μM, with a LOD of 10 fM which indicated that it could also be used in the detection of other toxins.

**Figure 5:**
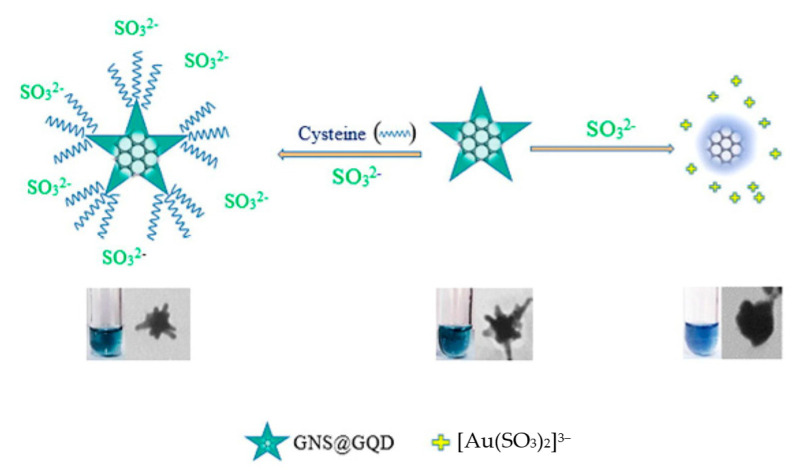
The etching reaction of AuNS@GQD with Na_2_SO_3_ and detection of cysteine based on anti-etching effect. Reprinted with permission from Ref. [61]. Copyright 2021 Elsevier.

The LSPR absorption peak could be changed not only by the variations of the distance between nanoparticles in the progress of aggregation or disaggregation but also by the changes in nanoparticles morphology during etching or growing. In contrast, the latter was much easy-to-operate, low-cost, and environmentally friendly. Recently, metal nanocomposites were proposed to enhance the sensitivity for detection via etching or growth mechanisms. For example, Zahra and co-workers [61] developed a gold nanostar@GQDs (AuNS@GQD) as a new colorimetric sensing platform for cysteine detection based on the anti-etching effect (Figure 5). According to the literature, Na_2_SO_3_ participated in the etching reaction of AuNS, which could induce the shape of AuNS to change to sphere-like nanoparticles, resulting in an obvious color change of the solution from light green to indigo. On the contrary, when cysteine was pre-added into the AuNS@GQD solution, it could interact with AuNS via Au-S bonding and protect AuNS from etching by Na_2_SO_3_. Therefore, the shape of AuNS and the LSPR band and color remained unchanged. This novel strategy with high sensitivity and selectivity was employed for the colorimetric detection of cysteine. Hallaj et al. [62] designed a dual colorimetric and fluorometric sensor by using N, P doped carbon dots and Ag nanoprisms (Ag NPrs) for the detection of 6-mercaptopurine (6-MP). In the colorimetric assay, I^−^ was used as an etching agent, which could change the morphology of Ag NPrs, resulting in the color change of Ag NPrs and the blueshift of the LSPR peak. However, in the presence of 6-MP, due to the protective effect of 6-MP on Ag NPrs, the color of the solution alerted to blueish, and the LSPR peak red-shifted. Because the Ag-S bond was much stronger than the Ag-I bond, 6-MP preferred to attach on the surface of Ag NPrs and inhibited the etching effect of I^−^. This dual colorimetric and fluorometric sensor had good linearity over a broad range (10–500 nM and 30–500 nM) and high sensitivity (4 and 10 nM). In addition, Hallaj’s group [63] also developed a dual colorimetric and fluorometric sensor based on fluorescein/Ag NPrs for the determination of pesticides. The mechanism of the dual sensor was based on the etching and protecting effect of S_2_O_3_^2−^ and mancozeb. In the presence of S_2_O_3_^2−^, the color of Ag NPrs solution changed from blue to purple and the LSPR peak blueshifted. This phenomenon was attributed to the etching effect of S_2_O_3_^2−^. When added into Ag NPrs solution, mancozeb can combine with Ag NPrs through Ag–S bond to protect Ag NPrs from etching; therefore, the color and LSPR peak remained unchanged. Zhao’s group [64] prepared a novel Au@AuAg yolk–shell heterogeneous nanostructure via Fenton-like reaction-mediated etching of Au, which was used for the determination of Co^2+^. In the presence of Co^2+^, due to the catalytic activity of Co^2+^, H_2_O_2_ was decomposed into O·_2_^−^ which can further etch the Au@AuAg yolk–shell heterogeneous nanostructure, accompanied by the changes in the position and intensity of LSPR peaks. The as-prepared sensor showed a good linear relationship between the intensity ratio and the concentration of Co^2+^ in the range from 1 to 100 nM with a LOD of 0.2 nM.

#### 2.1.3. DNA Nanomaterial-Based Colorimetric Sensors

DNA is a highly stable, easy-to-modify, and programmable biomolecule, which has been widely used as a sensing probe for target detection. Furthermore, the chemical synthesis of DNA oligonucleotides is low-cost with little batch-to-batch variation [65]. The mechanisms of DNA nanomaterial-based colorimetric sensors are all based on the affinity of DNA structures with targets which may cause the transformation of DNA nanostructures. The typical examples are G-quadruplex formed by metal ions (such as K^+^, Na^+^) with G-rich sequences [66,67], ion-bridged complexes formed between bases (such as T-Hg-T, C-Ag-C bridges) [68,69], DNAzymes, and so on [70]. Up to now, DNA-based nanomaterials have been reported and applied in the detection of metal ions [71,72,73], pathogenic microorganisms [74], viruses [75,76], and biomacromolecules [77,78,79].

##### G-Quadruplex-Based Colorimetric Sensors

G-quadruplex (G4) is a special DNA structure with flexible binding ability, which has been used for target recognition and signal transduction in biosensors. G4-based colorimetric sensors have attracted considerable attention for analytes detection due to their easy operation, high sensitivity, and low cost. G4 possesses many interesting properties such as binding metal ions, dyes, and porphyrin. In addition, G4 could also bind hemin to serve as a peroxidase-mimicking DNAzymes [80]. Therefore, G4-based colorimetric sensors have been extensively applied for metal ions detection [81,82], radioactive gas [83], small molecules [84], viruses [75,85], and proteins [78,86].

Metal ions could bond to the carbonyl oxygens in the G4 plane or between two planes, based on their size. Based on this property, many sensors for metal ions detection have been developed. In 2016, Liu’s group [87] screened a few sequences and designed a Tl^+^ biosensor based on a G4 sequence. As we know, Tl^+^ could induce DNA folding, whereas Au NPs had extremely high extinction coefficients and aggregation-induced color change. When DNA was folded by Tl^+^, the folded DNA can inhibit its adsorption on Au NPs, which were more easily aggregated, resulting in the color turning blue.

**Figure 6:**
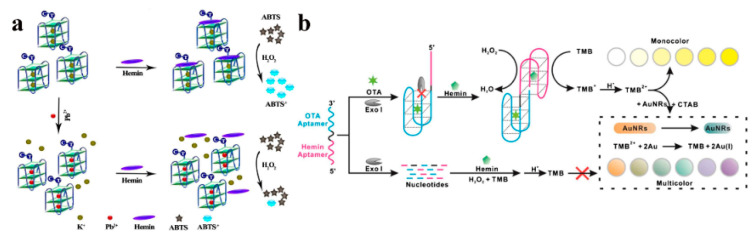
(**a**) Schematic diagram of G4 DNAzymes for Pb^2+^ detection based on the competition between K^+^ and Pb^2+^ stabilized G4. Reprinted with permission from Ref. [90]. Copyright 2019 Springer; (**b**) Mechanism assay for OTA detection by exonuclease-assisted multicolor aptasensor. Reprinted with permission from Ref. [84]. Copyright 2018 Springer.

To enhance the sensitivity and specificity of G4-based colorimetric sensors, hemin@G4 DNAzymes were developed. Hemin@G4 DNAzymes could serve as a mimic peroxidase to catalyze some colorless substrates to produce a color change such as ABTS [88] and TMB [89]. Based on the oxidation of ABTS, Ju and coworkers [90] designed G4/hemin DNAzymes (G4 DNAzymes) for sensitive colorimetric determination of Pb^2+^ (Figure 6a). The G4 DNAzymes were modified with a d [TC] sequence at the 3′ end and stabilized by K^+^. When Pb^2+^ was added, the K^+^ in the parallel G4 was replaced by Pb^2+^ to keep the parallel topology. However, the affinity between topology and hemin was decreased by the introduced Pb^2+^, which resulted in a decrease of DNAzyme activity for catalyzing the oxidation of ABTS to form a green dye. This simple visual sensor was performed without any amplification, and a good linearity ranging from 0.01 to 10 μM for Pb^2+^ concentration and a LOD of 7.1 nM was accomplished. Based on the oxidation of TMB, Fu et al. [84] developed an exonuclease-assisted multicolor aptasensor for ochratoxin A (OTA) visual detection (Figure 6b). In this colorimetric sensor, when OTA bound to a DNA sequence (AG4-OTA) consisting of a hemin aptamer and an OTA aptamer, its digestion could be halted by exonuclease I from the 3′-end of AG4-OTA. The remaining hemin aptamer could bind to hemin to form a G4-hemin DNAzyme, which showed peroxidase-like activity and could catalyze the oxidation of TMB by H_2_O_2_ to produce TMB^2+^ in an acid solution. TMB^2+^ could etch gold nanorods by oxidizing Au (0) into Au (I), presenting rainbow-like colors. This colorimetric sensor provided a multicolor platform for the visual detection of OTA.

As we know, microRNA (miRNA) and other biomolecules have become biomarkers for various diseases. Therefore, the development of miRNA sensors with low cost, simple operation, and high sensitivity has attracted increasing attention from scientists. In Lee’s previous work [91], they found GO could be combined with ssDNA via π–π interactions and hydrogen bonding, which could easily enrich the DNA probe. Based on this finding, Lee and coworkers [92] first reported a GO-based paper sensor for colorimetric sensing miRNA by the target-recycled signal amplification strategy. The paper-based miRNA sensor was developed in two steps: in the first step, G4 DNAzyme (Dz) was prepared and used to generate signals in colorimetric assays; in the second step, GO as a net (GONET) was employed to capture ssDNA probes via π–π interactions and hydrogen bonding, and the Dz/GO composite could be easily accumulated onto an intended spot on the paper, which can amplify Dz-catalyzed colorimetric reaction in the presence of hemin, ABTS, and H_2_O_2_.

##### DNA Tetrahedron-Based Colorimetric Sensors

DNA tetrahedron is a classical and simplest polyhedron, which was first reported by Turberfield and coworkers via one-step assembly [93,94]. DNA tetrahedron with a stable structure and uniform size is synthesized from four equal moles of well-designed 55-base oligonucleotides. Due to the high programmability of DNA tetrahedrons, they could be performed with artful design and combined with other materials. Especially, functional DNA tetrahedrons that mainly focus on four vertexes and six duplex-helix edges of the tetrahedron were developed to meet the demands of sensors, biomedical and other fields [95,96]. Up to now, DNA tetrahedron nanostructures have been used as a universal platform for the colorimetric detection of metal ions [97].

**Figure 7:**
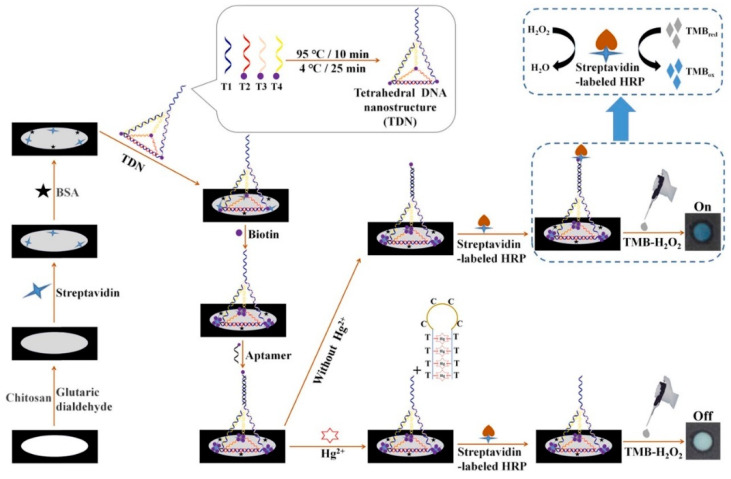
Schematic diagram of the fabrication procedures of DNA tetrahedron functionalized paper-based colorimetric sensor for Hg^2+^ detection. Reprinted with permission from Ref. [68]. Copyright 2022 Elsevier.

Due to the high toxicity and bioaccumulation, some metal ions have attracted considerable attention all over the world, which can cause severe environmental and health problems. Therefore, developing a highly sensitive and selective strategy for monitoring trace amounts of metal ions is essential. DNA tetrahedron nanostructure is a kind of rigid and stable scaffold, more importantly, its shape could be adjusted by the mechanical reconfiguration of the DNA strand at the arm of the tetrahedron using external stimuli [98]. In addition, the DNA tetrahedron structure is a suitable choice for carrying aptamer to enhance the sensitivity and specificity of the DNA tetrahedron-based sensors. For example, based on the strong and highly selective binding capacity of thymine–thymine base pairs toward Hg^2+^, Chen and coworkers [68] developed a DNA tetrahedron functionalized paper-based colorimetric sensor for the detection of Hg^2+^ (Figure 7). DNA tetrahedron served as a scaffold to anchor aptamer and improved the capture efficiency of this assay platform, because of its controllably specific orientation, low steric hindrance effect, and well-defined spacing. Biotinylated aptamer could bind with streptavidin-labeled HRP in the absence of Hg^2+^. While, in the presence of Hg^2+^, due to the higher binding affinity of T-Hg-T than T-A, biotinylated aptamer preferentially bound to Hg^2+^, which consequently resulted in the biotinylated aptamer releasing from DNA tetrahedron. Therefore, streptavidin-labeled HRP could not attach to the DNA tetrahedron functionalized paper-based sensor. After introducing TMB-H_2_O_2_, the obvious color signal change could be observed.

#### 2.1.4. Other Types of Colorimetric Sensors

**Figure 8:**
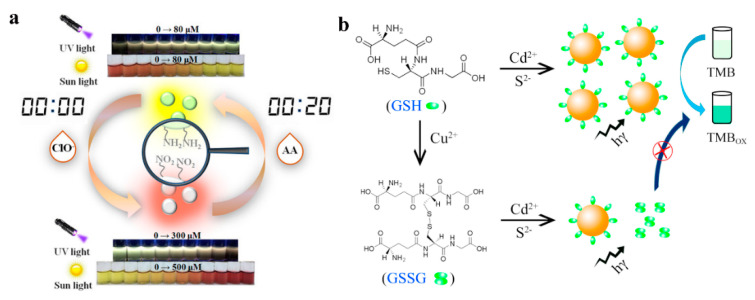
(**a**) The reversible sensing performance of CDs for ClO^−^ and AA in colorimetric and fluorescent mode. Reprinted with permission from Ref. [110]. Copyright 2019 ACS; (**b**) colorimetric sensor for Cu^2+^ based on the formation of quantum dots with photocatalytic activity. Reprinted with permission from Ref. [112]. Copyright 2017 Elsevier.

Quantum dots (QDs) belong to the zero-dimensional nanostructures which have attracted considerable attention due to their unique photochemical and photophysical properties. Carbon-based QDs and metal-based QDs are typical representatives of QDs. The size and crystal structure of QDs endowed them with many characteristics, making them widely used in various detection systems such as photoluminescence [99,100], photoelectrochemical [101,102,103], chemiluminescence [104,105], colorimetry [106], and electrochemiluminescence [107,108,109]. Among these detection systems, the colorimetric method was popular due to its convenience and visibility. Many colorimetric sensors have been developed for the analysis of biological and chemical samples. Chen et al. [110] designed carbon dots as a dual sensor for real time detection of hypochlorite (ClO^−^) and ascorbic acid (AA) in biological samples (Figure 8a). According to the colorimetric detection results, the absorbance at 509 and 548 nm increased with the addition of ClO^−^ but decreased with the addition of AA. This was assigned to the oxidation and reduction of surface functional groups of CDs by ClO^−^ and AA. Hemmateenejad and coworkers [111] synthesized molybdenum disulfide quantum dots (MoS_2_ QDs) to fabricate a new colorimetric sensor array for the recognition of aldehydes and ketones. The results demonstrated that the as-prepared colorimetric sensor could successfully distinguish eight aldehydes and ketones, and could accurately determine the content of formaldehyde in milk.

Furthermore, quantum dots can also be used as a photocatalyst for the oxidation of TMB to produce a colored product. Colorimetric sensors based on catalytic oxidation mechanisms possess various advantages of simple operation, low cost, and low background signal. It is worth noting that QDs-based catalytic oxidation reactions don’t require the introduction of an oxidant, hydrogen peroxide. For example, Zhang et al. [112] synthesized CdS QDs based on a simple thiol compound (glutathione, GSH) mediated method (Figure 8b). As we know, TMB is susceptible to photosensitized oxidation under the irradiation of UV light [113]. The as-prepared CdS QDs with photocatalytic activity were used as a catalyst which could accelerate the oxidation of TMB to produce a blue product without the addition of H_2_O_2_, under UV irradiation. During the sensing process, when Cu^2+^ was added into the CdS QDs and TMB solution, the thiol in GSH was oxidized by Cu^2+^, and GSH lost the stabilization ability for the growth of CdS QDs, leading to the decrease of the amounts of formed CdS QDs and the absorbance of ox-TMB. In addition, QDs also [114] exhibited photoreductive activity which could be used to fabricate a sensitive and selective colorimetric sensor for the detection of Cu^2+^. First, TMB could be oxidized to produce blue ox-TMB under UV irradiation. Secondly, when QDs were introduced into the TMB under the same irradiation condition, the oxidation of TMB was observed to be suppressed. This may be attributed to the photo-generated electrons from QDs, which could reduce part of ox-TMB back to TMB. In the presence of Cu^2+^, the absorbance of ox-TMB increased and the solution color intensified. This phenomenon illustrated that the photocatalytic ability of QDs was sensitive to the concentration of Cu^2+^. According to the literature, Cu^2+^ was easily attached to the surface of metal sulfide QDs and was quickly reduced to Cu^+^ under illumination [115,116]. Due to the formation of Cu_x_S on the surface of QDs, the original surface state of QDs may be changed, resulting in a decrease in the photoreductive ability. Amin and coworkers [117] synthesized positively charged gold quantum dots (Au QDs) directly from Au nanoparticles (Au NPs) by a hydrothermal process, which exhibited strong nanozymatic activity in the presence of SCN^−^. The results demonstrated that Au NPs showed higher catalytic ability than Au QDs in the presence of TMB and H_2_O_2_. This was probably because the high-temperature reaction changed the surface properties of Au QDs and made the nanozymatic activity of Au QDs disappear. When SCN^−^ was added into Au QDs solution, the nanozymatic activity was restored, due to the ·OH radical scavenging activity of SCN^−^. SCN^−^ played an important role in improving the sensitivity of this sensor by initiating the radical chain reaction and oxidizing TMB.

Carbon nanotubes (CNTs) have been used for the fabrication of colorimetric sensors, due to their huge surface area, excellent electrical conductivity, and easy modification. For example, a nanocomposite of zinc oxide nanoparticles and CNTs was prepared that can catalyze the oxidation of ABTS in the presence of H_2_O_2_ to produce green colored products. H_2_O_2_ is the oxidative product of cholesterol in the presence of cholesterol oxidase, therefore, the proposed colorimetric sensor could be used to detect cholesterol [118]. In addition, Wu and coworkers synthesized functional single-walled carbon nanotubes (SWCNTs) with different groups (–NH_2_, –COOH) to load hemin. Hemin was assembled to functional SWCNTs through π–π interaction. The results demonstrated that the peroxidase like activity of hemin has been enhanced by NH_2_@SWCNTs, which was much higher than hemin itself, SWCNTs @hemin, COOH-SWCNTs@hemin [119]. Moreover, graphitic carbon nitride (g-C_3_N_4_) nanosheets also have been employed to fabricate colorimetric sensors. For instance, Wu and colleagues synthesized boron- and phenyl-doped graphitic carbon nitride (BPCN NSs) to fabricate a colorimetric sensor for the detection of H_2_O_2_. BPCN NSs showed enhanced peroxidase-like activity and catalyzed the oxidation of TMB, ABTS and OPD in the presence of H_2_O_2_ [120].

### 2.2. Summary on the Colorimetric Sensors for Chemical and Biological Sensing Applications

Table 2 shows a list of sensor analytes and their respective probes. In the following section, we present the application of colorimetric sensors using the materials we discussed above as functional building blocks.

#### 2.2.1. Detection of Metallic and Non-Metallic Ions

Metallic and non-metallic ions could cause serious and urgent problems for public health and social development. Tremendous efforts have been made to investigate some efficient methods for monitoring these metallic ions. For instance, amine functionalized graphene oxide quantum dots (GOQDs) possessed good stability. GOQDs were used as the cross-linking agents and doped with poly(vinyl alcohol) (PVA) to prepare a solid sensing platform for optical detection of Fe^2+^, Co^2+^, and Cu^2+^, and the LOD was 1 × 10^−7^ M using UV-Vis spectroscopy [141]. Xian et al. proposed a novel [142], label-free colorimetric sensor for the detection of Pb^2+^ based on the acceleration of gold leaching by GO at room temperature. Compared with the colorimetric sensor without GO, the dissolution rate of gold in the Pb^2+^-S_2_O_3_^2−^-GO system is 5 times faster, and the LOD was around 0.05 μM with a linear range from 0.1 to 20 μM. Ahour et al. [143] reported a colorimetric method for the detection of pH and various cations by alizarine red S functionalized graphene quantum dots (GQD-ARS). GQD showed orange color in all pH values, whereas GQD-ARS exhibited different colors at different pH. For example, it showed yellow color at pH < 4 similar to ARS. When the pH increased from 4, the color of GQD-ARS changed from yellow to red, and the solution turned purple color at pH > 11.2. Due to the pH-related color of GQD-ARS, Fe^3+^, Co^2+^, Ca^2+^, As^3+^, Cd^2+^, Hg^2+^, Pb^2+^, Sn^2+^, Al^3+^, and Cr^3+^ were used to evaluate the colorimetric response of the as-prepared sensor. The results demonstrated that GQD-ARS had a great performance in the pH-related colorimetric detection of Co^2+^ and Fe^2+^ in the mixture of ions.

**Figure 9:**
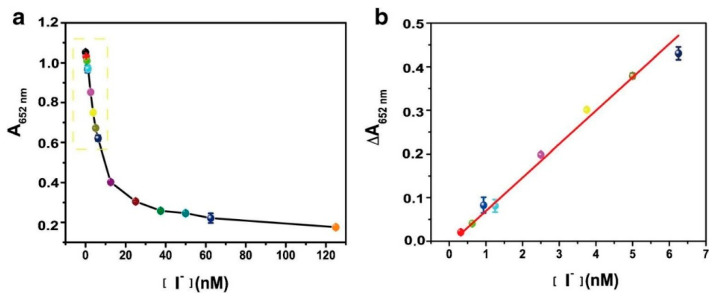
(**a**) Absorbance of TMB+H_2_O_2_+CC-PdNPs system at 652 nm in the presence of various concentrations of I^−^; (**b**) The linear relationship for I^−^ detection (ΔA_652nm_ = A_0_ − A). A_0_ and A represent A_652nm_ of TMB+H_2_O_2_+CC-PdNPs system and TMB+H_2_O_2_+I^−^+CC-PdNPs system, respectively. Reprinted with permission from Ref. [144]. Copyright 2020 Springer.

In addition to metal ions, colorimetric sensors could also be employed for the detection of non-metallic ions. For example, Hong et al. reported [144] a carboxylated chitosan-coated palladium (CC-PdNPs) nanozymes which was used to detect iodine ions. Because of the peroxidase-like activity of CC-PdNPs, it could catalytically oxide TMB to produce colored products in the presence of H_2_O_2_. As shown in Figure 9a, the absorbance of the TMB+H_2_O_2_+CC-PdNPs system at 652 nm decreased as the concentration of I^−^ increased. This method had good linearity when the concentration of I^−^ ranged between 0 and 6.25 nM, with a correlation coefficient of 0.995 and a LOD of 0.19 nM.

Zeng and coworkers [145] developed a specific colorimetric sensor for the detection of fluoride ions (F^−^) by triggering the intrinsic peroxidase-like activity of an AgPt-Fe_3_O_4_ nanozyme encapsulated in SiO_2_ shells. AgPt-Fe_3_O_4_ was encapsulated in SiO_2_ shells via the Stöber method, at the same time, the inherent peroxidase-like activity of AgPt-Fe_3_O_4_ was inhibited. However, F^−^ can exclusively etch SiO_2_ shells to expose the active sites of AgPt-Fe_3_O_4_ and recover its enzyme-like activity, thus causing color changes via the oxidation of TMB. The absorbance of the sensing system at 652 nm increased with increasing F^−^ concentration, accompanied by a concurrent color change from colorless to blue. There was a good linear relationship between ΔA_652nm_ and the concentration of F^−^ in the range of 50–2000 μM with a correlation coefficient of 0.988 and the LOD of 13.73 μM.

#### 2.2.2. Detection of Proteins

**Figure 10:**
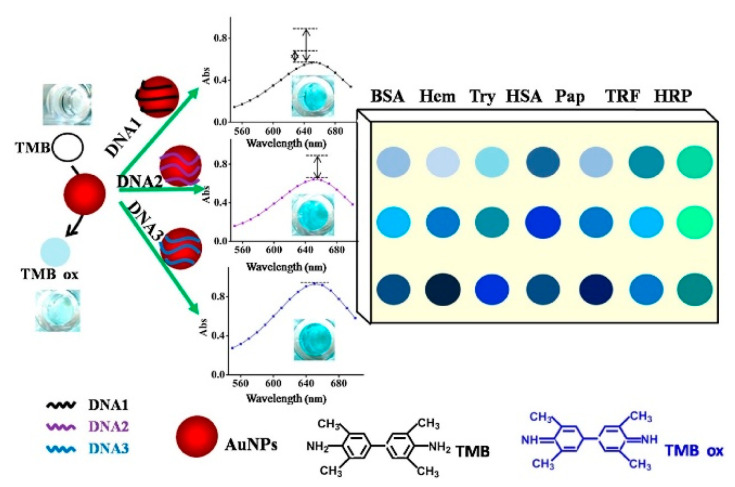
Schematic illustration of proteins discrimination by different AuNPs–DNA conjugates. Reprinted with permission from Ref. [146]. Copyright 2017 Elsevier.

Au NPs can be functionalized based on the adsorption capacity of AuNPs to oligonucleotides (DNA) to obtain high sensitivity and specificity of AuNPs-based materials. Liu and coworkers [146] designed a novel colorimetric sensor for protein discrimination based on the tunable catalytic activity of AuNPs–DNA conjugates. Different single-strand DNA (ssDNA) attached to the surface of AuNPs could enhance the peroxidase-like activity, leading to a highly sensitive colorimetric signal by catalyzing the oxidation of TMB. As shown in Figure 10, three ssDNA were used as the modifying reagents to tune the nanozymes-like activity of AuNPs. The results showed that the catalytic activity of AuNPs was enhanced to catalyze TMB in the presence of H_2_O_2_, accompanied by the significant improvement of the absorbance of ox-TMB at 650 nm. In addition, AuNPs–DNA conjugates could effectively distinguish various proteins and showed a different colorimetric response.

**Figure 11:**
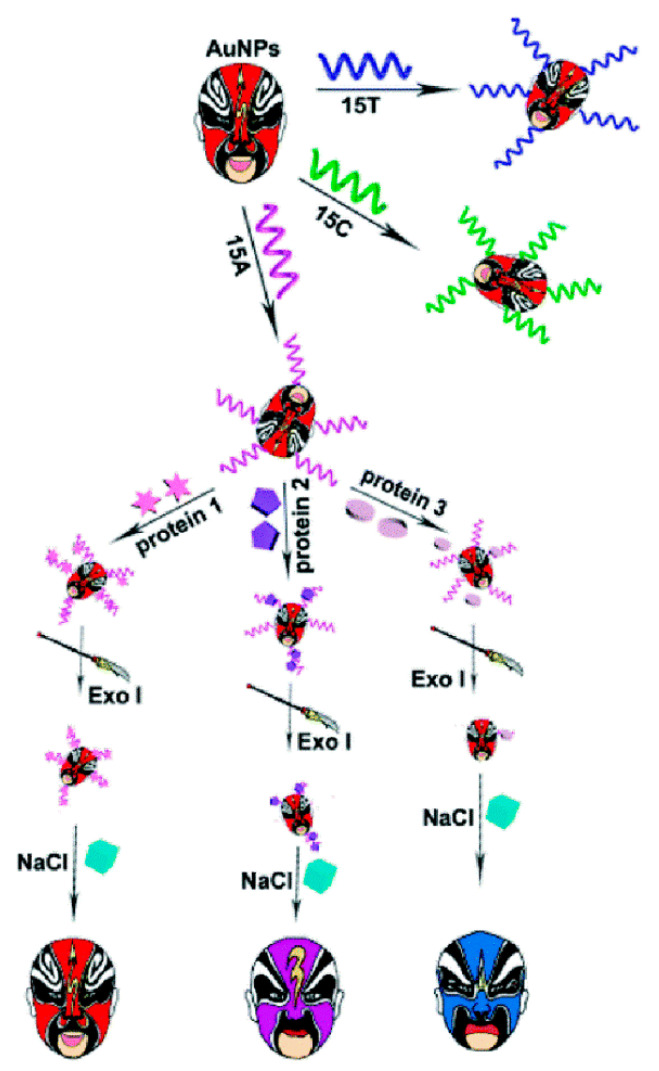
Schematic illustration of the design and fabrication process of the colorimetric sensor array for the identification of proteins. Reprinted with permission from Ref. [147]. Copyright 2019 RSC.

Based on the different aggregation behaviors of AuNPs–DNA conjugates in the presence of proteins and exonuclease I (Exo I) in the NaCl environment, Chen et al. [147] reported a colorimetric sensor assay for the distinguishing of different proteins (Figure 11). Due to the diverse affinities between different proteins and DNA immobilized on the AuNPs surface through the Au–S bond, the DNA–protein binding was resistant to the digestion of Exo I and protected AuNPs from aggregation in the high concentration of the NaCl solution. In the absence of proteins, DNA strands were digested by Exo I quickly, and the removed DNA would make AuNPs aggregate in the NaCl solution, leading to a color change from red to blue. However, in the presence of proteins, differential binding of proteins with DNA strands protected the DNA from being hydrolyzed by Exo I to diverse degrees, resulting in concomitant alteration in behaviors of AuNPs aggregation and various colorimetric responses. The proposed colorimetric sensor could correctly discriminate 15 proteins at 10 nM level through linear discriminant analysis with 100% accuracy. Furthermore, 35 unknown samples were used as the blind sample to evaluate the discriminant ability of the sensor, and 33 of the 35 unknown protein samples were recognized, affording a high recognition accuracy of 94.29%.

#### 2.2.3. Detection of Small Molecules

Various colorimetric sensors have been used for the high-performance detection of small molecules, including small-molecule drugs, small biomolecules, pollutants, and so on. For example, Li et al. [148] fabricated an efficient and convenient dual-response sensor based on the competition complexation of Cu^2+^ between g-C_3_N_4_ nanosheets and thiocholine for the detection of organophosphorus pesticide. Due to the peroxidase activity of Cu^2+^-g-C_3_N_4_, it was used to catalyze the oxidation reaction of TMB in the presence of H_2_O_2_. It was found that the catalytic activity of Cu^2+^-g-C_3_N_4_ was inhibited by AChE/ATCh. While in the presence of malathion (a model of organophosphorus pesticide), the catalytic activity of Cu^2+^-g-C_3_N_4_ was recovered owing to the inhibition of malathion on AChE. Therefore, the absorbance of TMB/H_2_O_2_ increased in the presence of malathion as a result of the inhibition of Cu^2+^ extraction from Cu^2+^-g-C_3_N_4_. The results showed that the linear range of the proposed colorimetric sensor was 7.5–50 nM, and the LOD was 1.497 nM. Hou’s group [149] developed an Au NPs-based colorimetric sensor for the detection of histamine as a spoilage monitor for distinguishing the lifetime and freshness of aquatic products. After the addition of histamine into the Au NPs solution, due to the electrostatic interaction between histamine and Au NPs, the interparticle distance between Au NPs was narrowed, which could change the size and dispersity of Au NPs, resulting in the color changes from wine-red to dark blue. Zhou and coworkers [150] also developed a novel sensing platform for the detection of histamine to evaluate fish freshness. Diamine oxidase (DAO) was immobilized on magnetic graphene oxide (MGO) by adsorption and covalent bonding, which exhibited a higher enzyme activity than the free DAO. Furthermore, MGO could also be used as an absorbent to separate histamine from fish samples, enhancing the sensing sensitivity and specificity. This colorimetric sensor was based on the AuNR etching reaction. After introducing histamine into the sensing system, it could be catalyzed by MGO-DAO to produce H_2_O_2_. Under the intervention of Na_2_MoO_4_, I^−^ could be oxidized to a strong oxidant I_2_ by H_2_O_2_, which could etch AuNRs longitudinally. This would lead to a series of color changes in fish samples solution with the different concentrations of histamine. These two colorimetric sensors could be used to semiquantitatively analyze histamine with the naked eye, but the colorimetric sensor fabricated by Zhou had a broader linear range than Hou’s.

#### 2.2.4. Detection of Gas

There are various gases presenting in the surrounding environment, some of which are colorless, flammable, possessing an irritating odor, and highly toxic. Therefore, it is necessary to detect them because of the potential threat to people’s health [151]. For example, Li et al. [152] developed a high-performance colorimetric sensor based on graphene/polystyrene sulfonate (GO/PSS) optical film for the detection of NO_2_ using a spin-coated assisted the layer-by-layer assembly method. The color of GO/PPS depended on the number of pairs, namely the thickness of GO/PPS.

The results indicated that humidity had a significant effect on the sensing performance. After introducing water, the absorbed water molecules could increase the interlayer distances of GO, and the sulfonate groups of PSS invert to the outside. Therefore, NO_2_ molecules entered into the negatively charged GO/PSS layers, resulting in a decrease in film thickness and a blue shift of the reflectance spectra. The as-prepared sensor showed satisfactory performance for NO_2_ detection and could be extended to practical applications. Yan and coworkers [153] fabricated a colorimetric and chemoresistive gas sensor based on Cu_2_O decorated Au nanochains (Cu_2_O-Au). Due to the strong chemical affinity of Cu_2_O for H_2_S and the LSPR efficiency of Au nanochains, this sensor exhibited great sensitivity and specificity for the naked-eye detection of H_2_S. According to the TEM image, EDX elemental mapping, and XRD of Cu_2_O-Au probe before and after H_2_S exposure, it could be concluded that Cu_2_O polyhedral were etched into smaller ones by S^2−^, and Cu_2_S may be formed. The color change of Cu_2_O-Au sol after adding S^2−^ was caused by the conversion of Cu_2_O into Cu_2_S. In addition, Cu_2_O-Au test strips were used to detect H_2_S gas and showed dramatic color changes from blue to yellow, and finally to brown after exposure with 2.5–100 ppm.

#### 2.2.5. Detection of Virus or Bacteria

Nanomaterials-based colorimetric sensors have been widely employed for the detection of bacteria and viruses. Au NPs-based hybrid materials exhibited a wide range of applications in virus detection. Park and coworkers [154] developed a capture immunoassay based on Ag-decorated AuNPs to enhance the peroxidase-like activity for real-time monitoring of the hepatitis E virus. Anti-HEV IgG antibody was used to conjugate Au nanoparticles (Ab-AuNPs) as the inner core, and in situ silver deposition on the surface of Ab-AuNPs as the outer shell. The synthesized nanocomposites with signal amplification function could entrap the target virus, whereas Ag-shell would be decomposed back to Ag^+^ by introducing TMB and H_2_O_2_. Based on this sensing mechanism, the as-prepared immunoassay could indirectly quantify the concentration of HEV and was employed for real-time monitoring of the HEV-infected monkeys. Wu and coworkers [155] fabricated a colorimetric sensor assay for the detection and identification of bacteria based on the specific affinity and electrostatic interactions between bacteria and Wulff-type 4-mercaptophenylboronic acid-mercaptoethylamine co-functionalized Ag NPs (MPBA-MA@AgNPs) at different pH. Various pH levels would significantly affect the aggregation or dispersion of Ag NPs. For example, Ag NPs preferred to disperse under neutral and alkaline conditions due to the specific affinity between cis-diol residues contained in carbohydrate-rich compositions on the bacterial cell surface and MPBA, resulting in the color of Ag NPs change from purple to yellow; in the acid condition, Ag NPs tended to aggregate due to the electrostatic interaction between positively charged MA and negatively charged bacteria, leading to the color change of Ag NPs from yellow to purple. Thus, bacteria could be detected or identified by the color of the sensing system at different pH conditions. This novel and simple colorimetric sensor could accurately distinguish the complex bacteria mixtures. Brennan et al. [74] proposed a reliable DNAzyme-based colorimetric paper for detecting *Helicobacter pylori* (*H. pylori*). For this purpose, a highly specific bacterium-activated RNA-cleaving DNAzyme was isolated by in vitro selection and was used as the sensor molecule. The colorimetric paper sensor was able to sensitively detect *H. pylori* in human stool samples and was stored at ambient temperature for at least 130 days.

#### 2.2.6. Detection of DNA/RNA

**Figure 12:**
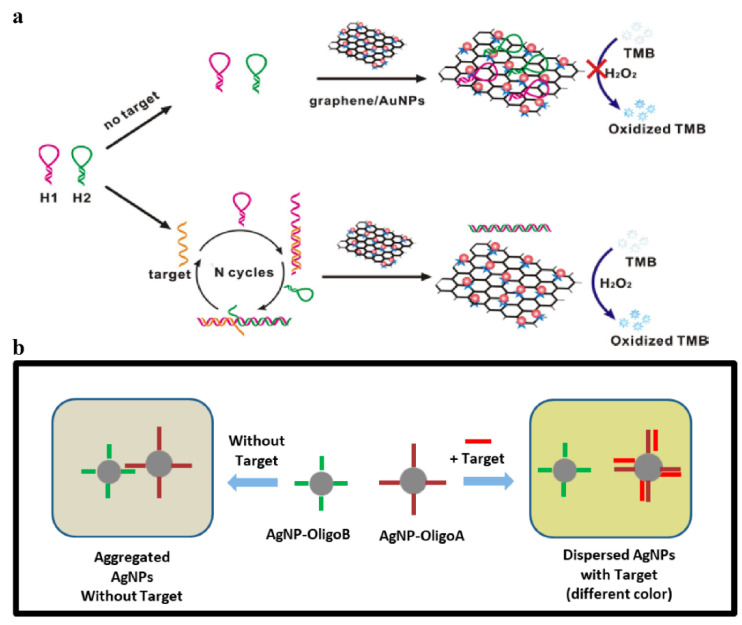
(**a**) Schematic of the label-free colorimetric platform for DNA by target-catalyzed hairpin assembly and the peroxidase-like catalytic of graphene/AuNPs hybrids. Reprinted with permission from Ref. [130]. Copyright 2016 Elsevier; (**b**) Schematic diagram of the sensing principle for miRNA detection by AgNPs-based assay. Reprinted with permission from Ref. [156]. Copyright 2021 MDPI.

DNA and microRNA (miRNA) are the potential non-invasive biomarkers for disease and injury. Many of the existing analytical methods for DNA/miRNA detection required complex procedures and expensive reagents. Therefore, it is necessary to develop a rapid and simple method for the detection of DNA/miRNA. He and coworkers [130] proposed a hybrid graphene/Au NPs platform based on target-catalyzed hairpin assembly for label-free detection of DNA. As shown in Figure 12a, in the absence of target DNA, H1, and H2 could not be assembled. Therefore, they were adsorbed by graphene/Au NPs, resulting in reducing the catalytic activity of graphene/Au NPs. However, in the presence of target DNA, the hairpin structure of H1 opened and hybridized with target DNA and H2 in turn, resulting in the formation of H1-H2 duplexes. H1-H2 duplexes could decrease the binding affinity with graphene/Au NPs, accompanied by a colorless to blue color change.

Vo-Dinh and coworkers [156] prepared a rapid and simple Ag NPs-based colorimetric detection of miR-21. Ag NPs were prepared and conjugated to two oligonucleotide sequences (oligo A and oligo B) specific to miR-21. As shown in Figure 12b, due to the hybridization between AgNP-oligoA and AgNP-oligoB nanoprobes, when mixed, the nanoprobes would aggregate. However, if the miR-21 target was added, it could competitively bind AgNP-oligoA to inhibit the aggregation of nanoprobes, accompanied by visible color change. On the basis of the sensing principle for miRNA, a smartphone-based device was developed to detect miRNA down to the nanomolar concentration.

## 3. Conclusions—Prospects and Challenges

In conclusion, we summarized the recent advances in the design, fabrication, and applications of colorimetric sensors. Based on all of the information above, it could be found that the sensing mechanism of colorimetric sensors mainly included the nanozymes-mediated chromogenic substrates/H_2_O_2_ reaction and the LSPR effect. Some nanomaterials, such as graphene and its derivatives, metal nanoparticles, DNA, and quantum dots exhibited excellent enzyme-mimicking activity and therefore could be employed for developing TMB/H_2_O_2_ reaction-based colorimetric sensors. In addition, a variety of doping materials were prepared to enhance the catalytic activity, leading to the improved sensitivity and specificity of the fabricated colorimetric sensors. Furthermore, Au NPs and Ag NPs could integrate with other nanomaterials to facilitate the LSPR change of the formed colorimetric sensors, thus inducing the variations of solution colors to achieve colorimetric detection. Therefore, through surface modification and hybridization with other nanomaterials, more functional colorimetric sensors could be developed to meet the distinct requirements. Colorimetric sensors can be used for biomarkers detection in clinical diagnosis, due to their advantages of naked-eye determination and rapid detection. However, biological samples are complex, and the concentration of biomarkers in biological samples are much lower. Therefore, how to develop promising tools with remarkable sensitivity and selectivity for biosensing is vital. At the same time, with the development of industry and agriculture, the problem of environmental pollution is particularly prominent. Colorimetric sensors with high sensitivity and easy accessibility can satisfy the needs of environmental monitoring. Moreover, to further enhance the safety of foods and water, different colorimetric sensors should be designed for detecting various pollutants.

A good sensor should not only have high sensitivity and selectivity, but also be economical and easy-to-operate. Graphene and its derivatives and other two-dimensional materials are not as economic as metal and metal oxide nanoparticles, due to their expense and difficulty to functionalize. DNA-based nanomaterials have relatively high detection efficiency, and the chemical synthesis of DNA oligonucleotides is low-cost. In addition, DNA tetrahedrons are usually employed as an inexpensive and easy tool for chemical and biological sensing. However, there are no systematic sequences of DNA templates, it needs to design DNA length and sequences rationally before constructing colorimetric sensors, which can increase the cost and difficulty of DNA-based colorimetric sensors. Currently, with the development of materials science, more and more low-cost and large-scale synthetic approaches have been developed, and the cost of colorimetric sensors will be decreased.

Although great improvements have been achieved in this promising research field, there are still challenges that should be faced: (1) Some nanomaterials, such as graphene, Au NPs, and Ag NPs tend to aggregate, which may affect the stability of the formed colorimetric sensors. (2) The dispersity of some nanomaterials is not good enough; therefore, the fabrication of colorimetric sensors could be influenced. To overcome this problem, adding water-soluble molecules into these nanomaterials could be a choice. (3) Some nanomaterials (e.g., DNA) exhibited low electron conductivity which would affect their catalytic activity. This problem might be solved by integrating these nanomaterials with some other particles with superior electron conductivity and a large specific surface area. (4) Although DNA-based nanomaterials showed excellent colorimetric sensor performance, they still face the risk of false positive signals, because of enzymatic degradation and interference of various non-target biomolecules. Some nanomaterials-assisted combinations or signal amplification strategies have been developed to solve this problem. With the development of material chemistry and analytical chemistry, we believe colorimetric sensors would contribute more to the field of chemical and biological sensing.

## Figures and Tables

**Table 1:** Colorimetric sensors based on graphene and its derivatives.

Materials	Analytes	Symbolic Parameters	Ref.
Graphene nanoribbons	Dopamine	This colorimetric sensor had a good linear in the concentration range of 0.1–50 μM with a LOD of 0.035 μM.	[16]
Heteroatom-doped graphene	Pesticides	The nanozyme sensor arrays exhibited linear response of five pesticides in the range of 5 to 500 μM.	[28]
L-cysteine functionalized graphene oxide	Hg^2+^	The colorimetric sensor was developed with CGO as an oxidase mimic for determination of Hg^2+^ with linear ranges of 0–200 μg/mL, and the LOD was 7.6 μg/mL.	[29]
Metal ions-TPA@GQDs	Thiols	—	[31]
Hemin–graphene nanosheets	Thyroglobulin	The gray intensity of the proposed paper-based sensor proved to be proportional to the concentration of thyroglobulin in the range of 5–100 ng/mL with a LOD of 1 ng/mL.	[32]
His@AuNCs/RGO	Nitrite	Under the optimized conditions, the proposed sensor showed a good linear correlation with nitrite concentration in the range of 10–500 μM with a LOD of 2 μM.	[33]
G/Fe_3_O_4_-Au NPs	Pb^2+^	Sensitive detection of Pb^2+^ was performed in the range of 1 to 300 ng/mL with the LOD was 0.63 ng/mL.	[34]
Oligonucleotide-GO/Au NPs	Hg^2+^	The absorbance value at the wavelength of 655 nm was linearly related with the concentration of Hg^2+^ in the range between 5.2 × 10^−9^ M and 1.2 × 10^−7^ M, and the LOD was 3.8 × 10^−10^ M.	[35]

**Table 2:** A list of sensor analytes and their respective probes.

Analytes	Probe	Ref.
Cu^2+^	Ag nanoparticles	[40]
L-histidine	Cu^2+^-modulated G-quadruplex	[77]
Hg^2+^	G-quadruplex	[82]
Glucose	Iron carbide nanoparticles	[121]
H_2_O_2_ and glutathione	Porphyrin modified ZnFe_2_O_4_/reduced graphene oxide	[122]
H_2_O_2_	Ag@TPE-SiO_2_ nanoparticles	[123]
Hg^2+^	Tetrahedral DNA	[124]
Pb^2+^	Dumbbell DNA	[125]
Melamine	Aptamer–DNAzyme conjunction	[126]
Cocaine	G-quadruplex	[127]
Puerarin	PtCu bimetallic nanoparticles deposited on PSS functionalized graphene	[128]
Explosive nitroaromatics	Au@Ag nanoparticles	[129]
DNA	Graphene/Au nanoparticles	[130]
Golgi protein 73	Reduced graphene oxide-carboxymethyl-hemin/platinum@palladium	[131]
Dopamine and glutathione	Graphene nanoribbons-silver nanoparticles	[132]
α-fetoprotein and prostate-specific antigen	Au/Bi_2_Se_3_	[133]
Cysteine	Pectinase protected Au nanoparticles	[134]
Norovirus	Ag/Au nanoparticles	[135]
Glucose-6-phosphate dehydrogenase	Ag nanoparticles	[136]
Kanamycin	Aptamer@terminal deoxynucleotidyl transferase	[137]
mRNA	DNA tetrahedron molecular beacon	[138]
HPV	Dextrin-stabilized Au nanoparticles	[139]
H_2_S and Hg^2+^	MnO_2_/multi-wall carbon nanotubes composite	[140]

## Data Availability

Data sharing not applicable.
